# Service delivery interventions to improve adolescents' linkage, retention and adherence to antiretroviral therapy and HIV care†

**DOI:** 10.1111/tmi.12517

**Published:** 2015-05-13

**Authors:** Peter MacPherson, Chigomezgo Munthali, Jane Ferguson, Alice Armstrong, Katharina Kranzer, Rashida A. Ferrand, David A. Ross

**Affiliations:** ^1^ Department of Public Health and PolicyUniversity of LiverpoolLiverpoolUK; ^2^ Department of Clinical SciencesLiverpool School of Tropical MedicineLiverpoolUK; ^3^ World Health OrganizationGenevaSwitzerland; ^4^ MRC Tropical Epidemiology GroupLondon School of Hygiene and Tropical MedicineLondonUK

**Keywords:** HIV, adolescents, antiretroviral therapy, retention, adherence linkage, systematic review, VIH, adolescents, thérapie antirétrovirale, rétention, lien d'adhésion, revue systématique

## Abstract

**Objectives:**

Adolescents living with HIV face substantial difficulties in accessing HIV care services and have worse treatment outcomes than other age groups. The objective of this review was to evaluate the effectiveness of service delivery interventions to improve adolescents' linkage from HIV diagnosis to antiretroviral therapy (ART) initiation, retention in HIV care and adherence to ART.

**Methods:**

We systematically searched the Medline, SCOPUS and Web of Sciences databases and conference abstracts from the International AIDS Conference and International Conference on AIDS and STIs in Africa (ICASA). Studies published in English between 1st January 2001 and 9th June 2014 were included. Two authors independently evaluated reports for eligibility, extracted data and assessed methodological quality using the Cochrane risk of bias tool and Newcastle–Ottawa Scale.

**Results:**

Eleven studies from nine countries were eligible for review. Three studies were randomised controlled trials. Interventions assessed included individual and group counselling and education; peer support; directly observed therapy; financial incentives; and interventions to improve the adolescent‐friendliness of clinics. Most studies were of low to moderate methodological quality.

**Conclusions:**

This review identified limited evidence on the effectiveness of service delivery interventions to support adolescents' linkage from HIV diagnosis to ART initiation, retention on ART and adherence to ART. Although recommendations are qualified because of the small numbers of studies and limited methodological quality, offering individual and group education and counselling, financial incentives, increasing clinic accessibility and provision of specific adolescent‐tailored services appear promising interventions and warrant further investigation.

## Abstract (fr)

**Objectifs:**

Les adolescents vivant avec le VIH font face à des difficultés plus importantes pour accéder aux services de soins VIH et ont des résultats de traitement pire que les autres groupes d’âge. L'objectif de cette revue était d’évaluer l'efficacité des interventions sur la prestation des services pour améliorer la liaison des adolescents à l'initiation de la thérapie antirétrovirale (ART) dès le diagnostic du VIH, la rétention dans les soins VIH et l'adhésion à l’ART.

**Méthodes:**

Nous avons systématiquement recherché sur les bases de données Medline, SCOPUS et Web of Sciences et des résumés de conférences de la Conférence Internationale sur le SIDA et de la Conférence Internationale sur le SIDA et les IST en Afrique (ICASA). Les études publiées en anglais entre le 1er janvier 2001 et le 9 juin 2014 ont été incluses. Deux auteurs ont indépendamment évalué les reports pour l’éligibilité, ont extrait les données et évalué la qualité méthodologique en utilisant l'outil de Cochrane pour le risque de biais et l’échelle de Ottawa‐Newcastle.

**Résultats:**

Onze études de neuf pays étaient éligibles pour l'analyse. Trois études étaient des essais contrôlés randomisés. Les interventions évaluées comprenaient: conseils et éducation individuelle et de groupe, soutien par les pairs, traitement directement observé, incitations financières et interventions visant à améliorer la convivialité des cliniques pour les adolescents. La plupart des études étaient de qualité méthodologique faible à modérée.

**Conclusions:**

Cette étude a identifié des preuves limitées sur l'efficacité des interventions de prestation de services pour soutenir la liaison des adolescents à l’ART dès le diagnostic du VIH, la rétention sous ART et l'adhésion à l’ART. Bien que les recommandations soient qualifiées à cause du petit nombre d’études et de la qualité méthodologique limitée, l'offre de l’éducation et les conseils individuels et de groupe, les incitations financières, l'accroissement de l'accessibilité de la clinique et la fourniture de services spécifiques adaptés aux adolescents apparaissent comme des interventions prometteuses et méritent une investigation plus poussée.

## Abstract (es)

**Objetivos:**

Los adolescentes viviendo con VIH se enfrentan a dificultades sustanciales en el acceso a los servicios de atención del VIH y tienen peores resultados frente al tratamiento que otros grupos de edad. El objetivo de esta revisión era evaluar la efectividad de intervenciones de prestación de servicios para mejorar, entre adolescentes, la vinculación desde el diagnóstico de VIH al inicio de la terapia antirretroviral (TAR), retención en los cuidados para el VIH y la adherencia al TAR.

**Métodos:**

Hemos realizado una búsqueda sistemática en bases de datos de Medline, SCOPUS y Web of Science y resúmenes de la Conferencia Internacional sobre SIDA e ITSs en África (ICASA). Se incluyeron todos los estudios publicados en inglés entre el 1 Enero del 2001 y el 9 Junio del 2014. Dos autores evaluaron, de forma independiente, los informes para determinar su elegibilidad, extraer datos y evaluar la calidad metodológica utilizando la herramienta Cochrane para la evaluación de riesgo de sesgo y la Escala Newcastle‐Ottawa.

**Resultados:**

Once estudios de nueve países eran elegibles para ser revisados. Tres estudios eran ensayos controlados aleatorizados. Las intervenciones evaluadas incluían: asesoramiento y educación individual y en grupo; apoyo de los compañeros; terapia de observación directa; incentivos financieros; e intervenciones para mejorar las facilidades para los adolescentes en las clínicas. La mayoría de los estudios eran de una calidad metodológica entre baja y moderada.

**Conclusiones:**

Esta revisión identificó una evidencia limitada sobre la efectividad de las intervenciones de prestación de servicios a la hora dar soporte a la vinculación entre el diagnóstico del VIH y el comienzo del TAR, la retención en el TAR y la adherencia al TAR entre adolescentes. Ofrecer educación y asesoramiento individual y grupal, incentivos financieros, aumentar la accesibilidad a las clínicas, y la prestación de servicios específicos y hechos a medida para adolescentes parecen ser intervenciones prometedoras. Sin embargo, aunque las recomendaciones son cualificadas, debido al número tan pequeño de estudios y a su calidad metodológica limitada, se requieren más estudios en el futuro.

## Introduction

An estimated 2.1 million adolescents (people aged between 10 and 19 years old) are infected with HIV 1, with the majority (85%) living in sub‐Saharan Africa 1. Adolescents are an underserved group in global and national responses to the HIV epidemic 2, 3, and, to date, global declarations, commitments and targets have failed to address their specific needs 1. Adolescents may be infected with HIV through sexual contact or exposure to unsafe injections (horizontal transmission); or *in utero*, during delivery, or whilst breastfeeding as an infant (vertically acquired) 4. About one‐third of infants who are infected with HIV will survive into adolescence without treatment 5. This means that large numbers of infants who were infected perinatally with HIV early in the epidemic before antiretroviral prevention of mother‐to‐child transmission was widely available are now surviving in adolescence due to increased availability of antiretroviral therapy (ART).

**Figure 1:**
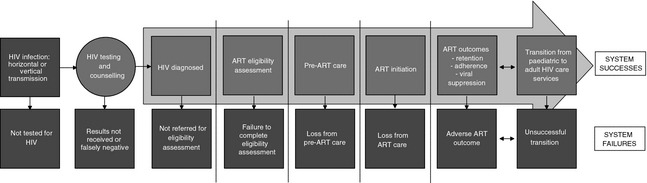
The adolescent‐focused HIV care pathway. Based on a figure by Kranzer and colleagues 79 (adapted).

Adolescents infected with HIV face particular challenges accessing effective care and achieving successful treatment outcomes (Figure 1). Adolescents undergo a period of rapid physical, emotional and behavioural change 3. They frequently face substantial socio‐economic problems, which may be accentuated if they are orphaned or looked‐after by social care services 11. Adolescents who were infected with HIV perinatally may have health and developmental problems including opportunistic infections, stunting, chronic lung disease and neuropsychiatric complications 2. There is little specific provision for adolescents within the health systems of most countries, with this group often falling into the gaps between paediatric and adult services. Problems related to the transition between paediatric and adult care are common 3 and include adolescents' fear of leaving supportive paediatric care, competing demands with higher education, starting employment and moving out of the family home, and insufficient proactive transition planning by health services, all of which may contribute to periods of worsened HIV care outcomes 6.

The few studies that have investigated HIV‐infected adolescents' progression through the HIV care pathway have generally found their outcomes to be poor and worse than for adults or young children. Rates of retention in care prior to antiretroviral therapy (ART) initiation have been reported to be substantially worse for adolescents than for adults 7. Once ART has been initiated, retention 8 and adherence to treatment are also poor, with a recent systematic review showing that, although studies used different measurement approaches, an average of only 62% of 12‐ to 24‐year olds achieved 95% or greater adherence 9.

Interventions to ensure adolescents' prompt linkage into HIV care and high levels of retention and adherence to ART could have substantial individual and public health benefits 10, 11, 12, but must be responsive to their unique needs. This study reviewed the effectiveness of service delivery interventions aimed at improving adolescents' linkage into HIV care and their subsequent retention and adherence to ART.

## Methods

### Study design

Systematic review.

### Inclusion and exclusion criteria

This review included studies published between 1st January 2001 and 9th June 2014. We sought studies that reported on the effect of service delivery interventions on adolescents' linkage from HIV diagnosis to ART initiation, retention on ART and adherence to ART. Both randomised controlled trials and non‐randomised studies (cohort studies, case–control studies, before and after and time‐series studies, and reports of routinely collected programme data) were included. Reviews, commentaries, editorials, case studies, case series, economic analyses, mathematical modelling studies and qualitative studies were excluded.

Adolescents were defined as study participants aged between 10 and 19 years. Studies were included if the majority (>50%) of study participants were adolescents. Studies that reported on mixed cohorts (young adults and adolescents, or older children and adolescents) were included if the mean or median age of the participants was between 10 and 19 years of age, or if the majority (>50%) of participants were aged between 10 and 19 years. In other studies, where the effect of interventions on participants with a wider range of ages was investigated, we included studies where age‐disaggregated data relevant to adolescents were reported.

Service delivery interventions 13 were defined to be interventions provided through health systems or health providers with the objective of improving adolescents' linkage from HIV diagnosis to ART initiation, retention on ART or adherence to ART. Studies that reported exclusively on the effect of one or more specific clinical treatment options (such as specific ART regimens or combinations) were excluded.

Completion of steps on the HIV care pathway between HIV diagnosis and initiation of ART was defined to include any of completion of assessment of ART eligibility (clinical staging, CD4 count measurement); completion of educational sessions and training; continuous collection of prophylactic therapy; and initiation of ART. In line with WHO recommendations on terminology 14, we defined retention on ART as continuous engagement in ART treatment, with retained participants being adolescents who attended at least one scheduled treatment appointment within the last 90 days. Adherence to ART was defined as regularly taking all antiretroviral medications that made up the prescribed ART regimen in the correct dose and at the correct time. Studies used a variety of outcome measures to define these outcomes of interest. Where possible, we used WHO‐recommended definitions for each of the three key outcomes (linkage to ART, retention, adherence). Where other outcome measures were used, we report outcomes as defined by the study's authors.

We placed no restrictions on countries studied. Only studies published in English were included.

### Search strategy

We searched the Medline (via PubMed), SCOPUS and Web of Sciences databases following a pre‐defined search strategy included within our published study protocol registered in the University of York database for Prospectively Registered Systematic Reviews in Health and Social Care (PROSPERO) 15. Search terms and full search strategies are shown in Figure S1. We also searched the online databases of conferences abstracts from the International AIDS Conferences (2006–2014) and the International Conference on AIDS and STDs in Africa (Dates 2008–2013). The bibliographies of selected studies were reviewed by hand to identify studies that may have been missed by our search strategy.

### Selection of studies

After exclusion of duplicate studies, two reviewers (PM and CM) independently screened the title and abstracts of all retrieved studies to identify studies eligible for full text review. Discrepancies were resolved by discussion between the reviewers, with arbitration by a third reviewer (DR) where agreement could not be reached.

A data extraction form, which had been pilot tested on a sample of manuscripts, was completed independently by two reviewers (PM and CM) for all studies selected for full text review. Reviewers recorded the presence of inclusion or exclusion factors, and if the study was eligible for inclusion, the following information was recorded from each study: year of study; study setting and population; description of study design and intervention(s) investigated; and outcome measures assessed. For each study with data available, the proportion (and 95% confidence interval [CI]) of adolescents completing steps on the HIV care pathway, retained on ART and adherent to ART, were recorded. Where these data were not available, study‐reported outcomes were extracted. Where a control or comparison group was evaluated, the relative and absolute effect of the intervention and 95% CIs were recorded, where available. If duplicate or partially duplicated results were presented in more than one study, we extracted data from both studies and included only the study with the most complete data.

Two reviewers (PM and CM) undertook an assessment of methodological quality for each study. For randomised controlled trials, the Cochrane Collaboration's Tool for Assessing Risk of Bias was used 16. For non‐randomised studies, a modified version of the Newcastle–Ottawa Scale was used 17. The Newcastle–Ottawa Scale was modified by removing items relating to procedures for selection of the non‐exposed cohort, as most studies did not include a control group. Given the possibility of highly heterogeneous study designs, an additional item was added to assess the presence any other potential sources of bias such as the size of the study and reported fidelity to study interventions. Results were compared and a consensus overall risk of bias judgement was made for each study. Randomised studies were graded as providing good (score 5–6), moderate (score 3–4) or low quality of evidence (0–2). Non‐randomised studies were graded as providing good (score 7–10), moderate (score 4–6) or low quality of evidence (score 0–3). An overall judgement of ‘unclear risk of bias’ was made where there was inadequate information provided by the study report.

Because we anticipated that studies would come from a wide variety of settings and populations, and report on a range of different interventions, we expected that there would be a high degree of heterogeneity. We therefore did not plan to attempt to estimate summary measures of effectiveness for interventions, but would have done so if the review indicated this to be appropriate.

## Results

### Study characteristics

The search identified 3138 unique abstracts, from which 3070 were excluded after review of title and abstract (Figure S2). Seventy‐four manuscripts were reviewed in full, of which 11 studies met the review's inclusion criteria 18, 19, 20, 21, 22, 23, 24, 25, 26, 27, 28. The main reason for exclusion was age (41/63, 65% of exclusions), with studies including a majority of participants aged <10 years old or >19 years old, and not reporting age‐disaggregated data to allow extraction data for all or part of the 10‐ to 19‐year age group (Table S2 – reasons for exclusion).

Characteristics of the 11 included studies are summarised in Table S1. Studies were conducted between 1998 and 2012. Six studies were from the USA 18, 20, 23, 26, 27, 28, one from each of the UK 21, France 22, South Africa 19 and Thailand 24, and one study was a multicentre study conducted in Kenya, Mozambique, Rwanda and Tanzania 25. Three studies were randomised controlled trials 18, 19, 26, and eight were non‐randomised studies with a variety of study designs, including prospective 21, 22, 24 and retrospective cohort studies 20, 23, and reports of routinely collected programme data 25, 27, 28.

The number of participants included in studies ranged between 9 23 and 57 038 25. The proportion of adolescent study participants that were male ranged between 25% 21 and 60% 20. There was considerable variation between studies in definitions of ‘adolescence’, ‘youth’ and ‘young adult’. Three studies specifically included key populations, including men who have sex with men 20, 28 and injecting drug users 20, 26.

Study settings comprised HIV care clinics (*n* = 9 studies 18, 19, 20, 21, 22, 24, 25, 26, 27, two of which described clinics as being specialist or dedicated adolescent centres 18, 21), a children's hospital 23, and an inpatient rehabilitation facility for children and adolescents affected by HIV 28. Two distinct groups of study participants were included in the different studies: some only included HIV‐infected adolescents meeting defined criteria for treatment failure or suboptimal adherence 21, 23, 24, 26, 27, 28, whilst others included HIV‐infected adolescents, selected irrespective of treatment success/failure or adherence 18, 19, 20, 22, 25.

**Figure 2:**
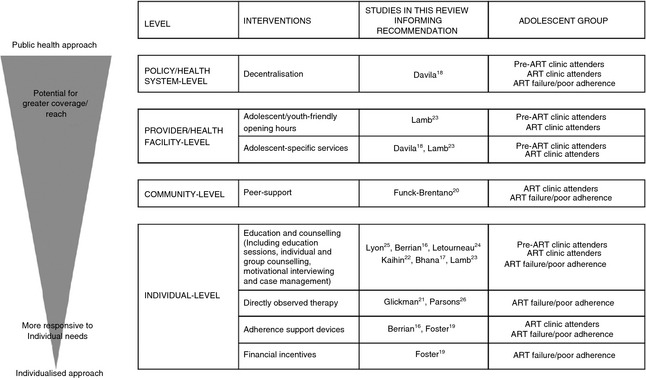
Typography of interventions to improve adolescents' linkage, retention and adherence to ART. Where studies evaluated interventions consisting of more than one component, or complex multifaceted interventions, they are listed more than once in the figure.

In the eleven studies included in the review, a total of eight interventions were evaluated. We classified interventions as having been applied at the individual‐, community‐, provider and health facility‐, and policy and health system levels (Figure 2).

Interventions applied at the individual level (education and counselling, directly observed therapy, adherence support devices and financial incentives) and community level (peer support) tended to be intensive, applied in multiple sessions over weeks to months, and were personally tailored to be responsive to the needs of individual adolescents. In contrast, interventions applied at the provider and health facility‐level (adolescent/youth friendly clinic opening hours, provision of adolescent‐specific services) and policy and health system level (decentralisation) emphasised individual patient needs less, but had greater potential to reach a larger number of adolescents and used fewer resources than individual‐level approaches.

### Linkage to HIV care and ART

Only one study evaluated interventions to improve adolescents' linkage to HIV care and ART 25 (Table 1a). Routinely collected clinic and patient data from a multisite study in four countries in sub‐Saharan Africa were used to evaluate the effect of availability of clinic interventions on pre‐ART attrition. At the clinic‐level, two sets of interventions were either available or not available: adolescent‐targeted services (defined as clinics that had dedicated adolescent clinics and opening hours, peer educators, or support groups) and services likely to be used by youth (clinics with screening for sexually transmitted infections, provision of condoms and hormonal contraceptives, and education). In adjusted analysis, none of the available clinic adolescent‐targeted services likely to be used by youth were associated with risk of pre‐ART attrition.

**Table 1:** Summary of findings – interventions to (a) improve adolescents' retention in pre‐ART care and linkage to ART, (b) improve adolescents' retention on ART and (c) improve adolescents' adherence to ART

Study	Outcome evaluated	Intervention or exposure	Number receiving intervention	Number with missing outcome	Number with outcome of interest (%)	Comparison	Number receiving comparison	Number with missing outcome	Number with outcome of interest (%)	Relative effect (95% CI) of intervention	*P*‐value
(a)											
Lamb 25, a	Cumulative incidence of attrition from pre‐ART care after 1 year	Adolescent‐specific clinic opening hours	NR^b^	NR	NR	No specific adolescent clinic opening hours	NR^b^	NR	NR	1.06 (0.89–1.27)^¥^	NR
(b)											
Studies including all HIV‐infected adolescents attending clinics											
Davila 20	Proportion of participants with at least 3 clinic visits during 1 year follow‐up	a. Centralised and enhanced youth support activities	48	NR	31 (64.5%)	b. Centralised care with youth multidisciplinary clinics	90	NR	51 (56.7%)	(a *vs*. b): 1.18 (0.55–2.53)^c^	0.68
						c. Decentralised care only	36	NR	11 (30.6%)	(c *vs*. b): 0.42 (0.17–1.03)^c^	0.06
	Proportion of participants with no gaps in care of >180 days during 1 year follow‐up	a. Centralised and enhanced youth support activities	48	NR	46 (95.8%)	b. Centralised care with youth multidisciplinary clinics	90	NR	72 (80.0%)	(a *vs*. b): 5.56 (1.20–25.0)^c^	0.03
						c. Decentralised care only	36	NR	30 (83.3%)	(c *vs*. b): 1.37 (0.46–4.17)^c^	0.57
Lamb 25	Cumulative incidence of attrition from ART after 1 year	Adolescent‐specific clinic opening hours	NR^b^	NR	NR	No specific adolescent clinic opening hours	NR^b^	NR	NR	1.48 (1.05–2.08)^¥^	NR
(c)											
Studies including HIV‐infected adolescents attending clinics, who had previous problems with retention or adherence, or poor treatment outcomes											
Foster 21	1. Change in median HIV viral load between baseline and 12 months	Financial incentives linked to HIV viral load results, motivational interviewing, provision of adherence support devices	11	0 (0%)	Median at baseline: 12 900 copies/ml Median at 12 months: 105 copies/ml	No comparison group	–	–	–	–	–
	2. Change in median HIV viral load between baseline and 24 months		11	1 (9.1%)	Median at baseline: 12 900 copies/ml Median VL at 24 months: <50 copies/ml		–	–	–	–	–
Glikman 23	1. Mean change in HIV viral load from pre‐DOT to completion of DOT	Inpatient DOT for 7 days, supported by education from physicians, nurses, nutrition specialists and social workers	13 admissions for 9 patients	2 (15.4%)	Mean change (SD): −0.8 (0.55)	No comparison group	–	–	–	–	0.04
	2. Change in mean HIV viral load from pre‐DOT to 6‐months post‐DOT		13 admissions for 9 patients	NR	NR	No comparison group	–	–	–	–	NR (‘not significant’)
Kaihin 24	Proportion of participants achieving >95% adherence 8 weeks after last empowerment group session	Empowerment building weekly group sessions	23	0 (0%)	Baseline: 88.0% Follow‐up: 97.4%	NR	23	0 (0%)	Baseline: 88.0% Follow‐up: 89.9%	NR	NR^∑^
Letourneau 26	Between‐group comparison of rate of change in mean ‘medication adherence score’^d^ over 9 months	Multisystematic therapy provided for 6‐months	20	NR	NR	Usual care with motivational interviewing and financial incentives for attendance	14	NR	NR	OR: 0.93 (no CI presented)^e^	0.693
Lyon 27	Before and after comparison of change in responses to National Institute of Health Adherence to Medication Questionnaire	12‐week multidisciplinary family curriculum	18	0 (0%)	Skipped at least one dose in the past 2 weeks: Before: 78%, After: 36% Skipped at least one dose yesterday: Before: 50%, After: 12% Skipped at least one dose in the past 2 days Before: 43%, After: 18%	No comparison group	–	–	–	–	–
Parsons 28	1. Change in mean HIV viral load (log_10_) between admission and discharge	DOT provided with multidisciplinary inpatient care	19	0 (0%)	Admission: 5.76 Discharge: 4.77	No comparison group	–	–	–	–	<0.001
	2. Change in mean HIV viral load (log_10_) between admission and 6 months after discharge	DOT provided with multidisciplinary inpatient care	19	0 (0%)	Admission: 5.76 6 months: 5.05	No comparison group	–	–	–	–	0.004
Studies including all HIV‐infected adolescents attending clinics											
Berrian 18	1. Mean difference in rate of collection of monthly prescribed ART, averaged over 3 months and ascertained from pharmacy records	8 structured nurse home visits over 3 months, with provision of education and adherence support devices	20	1 (5.0%)	NR	Clinic‐based adherence advice with provision of adherence support devices	19	2 (10.5%)	NR	NR	0.002
	2. Mean difference in self‐reported adherence (not further defined)	8 structured nurse home visits over 3 months, with provision of education and adherence support devices	20	NR	Mean difference (SE) in adherence score (0–3 months): +2.78 (0.88)	Clinic‐based adherence advice with provision of adherence support devices	19	NR	Mean difference (SE) in adherence score (0–3 months): +0.2 (0.96)	NR	0.07
Bhana 19	Change in ‘missed last ART dose’^f^ using paediatric AIDS clinical trials group adherence questionnaire assessed between baseline and 3.5 months	6 sessions of family group therapy, with cartoon aids	33	NR	Baseline: 3.71 Follow‐up: 4.81	Intervention delivered following completion of study	32	NR	Baseline: 4.79 Follow‐up: 4.36	NR	0.05
Funck‐Brentano 22	Change in proportion of participants with undetectable HIV viral load (<200 copies/ml) between baseline and 24 months	Group 1: 90‐min group therapy and peer support session once every 6 weeks for 26 months	10	0 (0%)	Baseline: 3 (30%) Follow‐up: 8 (80%)	Group 2: declined to participate	10	1 (1%)	Baseline: 3 (33%) Follow‐up: 5 (56%)	NR	NR
						Group 3: lived too far to participate	10	0 (0%)	Baseline: 5 (50%) Follow‐up: 5 (50%)	NR	

NR, Not reported; CI, confidence interval; SE, standard error; DOT, directly observed therapy; OR, odds ratio.

a Study included under linkage to ART and retention on ART.

b Total of 3794 participants aged 10–14 years and 53 244 aged 15–24 years in the study as a whole.

c Adjusted odds ratio.

d Medication adherence score' comprised of mean response to 3 items (‘per cent of days that any medications were taken, whether all doses were taken, and whether medication was taken according to instructions’).

e Odds ratio for difference between groups in rate of change in mean medication adherence score.

f Not clear what scores are measuring from manuscript.

### Retention on ART

Two studies 20, 25 investigated the effect of interventions to improve adolescents' retention on ART (Table 1b). Interventions evaluated included a multidisciplinary adolescent clinic staffed by adolescent physicians, care managers and social workers 20; decentralisation of care (defined by authors to be no provision of specific adolescent‐targeted services) 20; peer support groups 20, 25; and adolescent‐specific clinic opening hours 20, 25.

In an ecological study in one US HIV clinic, adolescents who had access to multidisciplinary adolescent clinics with individualised support were significantly less likely to have gaps in care compared to adolescents treated when these services were not available 20. Surprisingly, in evaluation of programmatic data from four sub‐Saharan countries 25, the cumulative incidence of attrition from ART 1 year after starting treatment was significantly higher amongst clinic attenders where adolescent‐specific clinic opening hours were available. Availability of peer support groups was associated with lower risk of attrition from ART in univariate analysis, but was not examined in adjusted models 25.

### Adherence to ART

In total, five interventions to improve adolescents' adherence to ART were evaluated, including counselling and education; use of adherence support devices; financial incentives; peer support; and directly observed therapy (Table 1c).

Seven studies evaluated counselling and education interventions 18, 19, 21, 22, 24, 26, 27. Counselling and education were offered to adolescents and their carers by clinic staff and researchers in a variety of ways, including as individuals sessions with a single counsellor 18; family sessions with a single counsellor 21; multidisciplinary sessions with an adolescent and/or their carers 19, 26, 27; and group counselling sessions 22, 24. Some studies used established counselling curricula used in previous studies such as multisystemic therapy 26 and motivational interviewing 21, whilst others used open‐ended and bespoke counselling and education techniques 18, 19, 22, 24, 27.

In studies of counselling and education, although participant groups, settings and content of interventions varied considerably, there was some evidence of effectiveness, although caution is required due to small study sizes and methodological issues. In a non‐randomised study conducted amongst adolescents attending two HIV clinics in Roi‐Et Province in Thailand, adolescents who received group counselling aimed at improving empowerment had greater improvements in ART adherence compared to adolescents who did not receive group counselling 24. Adolescents with adherence difficulties attending an urban clinic in the USA who participated in 12‐week multidisciplinary family counselling sessions showed substantial reductions in missed ART doses at the end of the programme 27.

Peer counselling (group counselling sessions where adolescents patients provided motivation and support to each, often facilitated by a clinic staff member or researcher) was investigated in one study of adolescents attending a hospital outpatient clinic in France 22. Groups were not randomly allocated to receive peer support or control interventions and numbers were small. After 2 years of follow‐up, the proportion of participants achieving viral suppression was higher amongst adolescents who received peer counselling.

In a study from a UK specialist centre, variable amounts of financial incentives linked to achievement of HIV viral load results were offered to adolescents with complex ART histories and previous multiple treatment interruptions 21. Results were promising, but limited by small numbers and lack of comparison groups.

In two studies, adherence support devices (for example, medication boxes and beepers) were provided to individuals as part of packages of interventions, including educational and counselling 18, or motivational interviewing and financial incentives 21. It is therefore difficult to distinguish the effects of adherence support devices from other interventions provided.

Two studies evaluated inpatient directly observed therapy (DOT) provided to adolescents with previous adherence difficulties or virological failure 23, 28. Both studies had small numbers of participants, and although participants' rates of virological failure showed improvements in the short term (by the end of completion of DOT), results were not sustained at 6 months after completing DOT.

### Quality and risk of bias assessment

The methodological quality of included studies was assessed as weak (summarised in Table 2 and reported in detail for each study in Table S3). All three of the included randomised controlled trials had methodological and reporting factors that meant they were classified as being at low methodological quality. Notably, none of the trials completely followed the CONSORT statement guidelines for reporting randomised controlled trials. Particular concerns were noted with procedures for randomisation and allocation (either at high risk of bias or not reported) 18, 19, 26, protocol‐driven consistency in application of interventions to participants 18, 19, 26 and selective reporting of outcomes 19, 26.

**Table 2:** Methodological quality and risk of bias assessment for included studies

Randomised controlled trials							
Domains	Sequence generation		Blinding of participants and outcome assessors	Incomplete outcome data	Selective outcome reporting	Other sources of potential bias	Overall quality of study
Signalling questions	Was the allocation sequence adequately generated?	Was allocation adequately concealed?	Was knowledge of the allocated intervention adequately prevented during the study?	Were incomplete outcome data adequately addressed?	Are reports of the study free of suggestion of selective outcome reporting	Was the study free of other problems that could put it at a high risk of bias?	
Adolescents' completion of steps of HIV care pathway							
No studies							
Adolescents' retention on antiretroviral therapy							
No studies							
Adolescents' adherence to antiretroviral therapy							
Berrian 18	Yes	No	No	Yes	Unclear	No	Low
Bhana 19	Unclear	Unclear	Unclear	No	No	No	Low
Letourneau 26	No	No	No	No	No	No	Low

Non‐randomised studies											
Domains	Participant selection		Exposure to intervention		Comparability	Assessment of outcomes				Other potential sources of bias	Overall quality of study
	Were participants selected to be representative of the target population	Were there clear participant selection criteria (for both exposed and control group if relevant), avoiding inappropriate exclusions?	Was a comparator/control group assessed?	Was the intervention being studied applied consistently to all eligible participants?	Were potential confounders identified and appropriately adjusted for?	Were procedures for assessment of outcome sufficient to satisfy confirmation of presence of condition of interest?	Was follow‐up long enough for outcome to occur?	Were incomplete outcome data adequately addressed?	Are reports of the study free of suggestion of selective outcome reporting?	Was the study free of other problems that could put it at a high risk of bias?	
Adolescents' completion of steps of HIV care pathway											
Lamb 25	Yes	Yes	Yes	Unclear	Yes	Yes	Yes	Unclear	Yes	No	Good
Adolescents' retention on antiretroviral therapy											
Davila 20	Unclear	Yes	Yes	No	No	Unclear	Yes	No	No	No	Good
Lamb 25	Yes	Yes	Yes	Unclear	Yes	Yes	Yes	Unclear	Yes	No	Good
Adolescents' adherence to antiretroviral therapy											
Foster 21	Yes	Yes	No	Yes	No	Yes	Yes	Yes	Yes	Yes	Good
Funck‐ Brentano 22	No	No	Yes	Yes	No	No	Yes	Unclear	No	No	Low
Glikman 23	No	Yes	No	Unclear	No	Yes	Yes	Yes	No	No	Moderate
Kaihin 24	Unclear	No	Yes	Unclear	No	No	Yes	Yes	Yes	No	Moderate
Lyon 27	No	No	No	Unclear	No	Yes	Yes	Yes	Yes	No	Moderate
Parsons 28	Yes	Yes	No	No	No	No	Yes	Yes	Yes	No	Moderate

None of the randomised controlled trials reported calculations to determine optimal sample sizes for detection of effects, and all three sample sizes were small, ranging from 34 26 to 65 19 randomised participants. The relative or absolute effects of interventions were only reported by one randomised controlled trial 26, and no trials reported confidence intervals around effect estimates, with studies instead usually reporting only *P*‐values for between‐group comparisons. The possible effects of confounding variables were not adequately considered in any of the randomised controlled trials identified by the review. Handling of missing exposure and outcome data was also problematic in two studies 19, 26.

Of the eight non‐randomised studies included, three studies were assessed as being of good methodological quality 20, 21, 25, four studies were of moderate quality 23, 24, 27, 28, and one was of low quality 22. Common methodological problems included small sample sizes 20, 21, 22, 23, 24, 27, 28; inconsistency in application of interventions to participants 20, 28; failure to identify and appropriately adjust for potential confounding variables 20, 21, 22, 23, 24, 27, 28; lack of comparison groups 20, 21, 23, 27, 28; and selective outcome reporting 20, 22, 23. In the studies reporting on interventions to improve adolescents' adherence to ART, particular concerns included the use of non‐standard and non‐validated measures of adherence.

## Discussion

The main findings from this systematic review were that, despite having worse HIV care outcomes than other age groups 1, few studies that have evaluated interventions to support adolescents' linkage, retention and adherence to ART have been reported. As we move towards an era of universal treatment for HIV 10, the clinical and public health benefits of widening access to ART for adolescents will not be realised until cost‐effective and sustainable service delivery interventions are widely implemented. The current evidence base from research on how best to do this is very weak. Although there are hints that some interventions may be effective, there is an urgent need for more rigorous, and larger, studies to clarify the effectiveness and replicability of potentially encouraging interventions.

We identified only 11 studies over a period of 13 and a half years, emphasising that the particular needs of adolescents living with HIV have not been widely studied 5. Moreover, the majority of studies were conducted in the USA (*n* = 6) or Europe (*n* = 2), with only three studies conducted in countries with generalised HIV epidemics, where the majority of the world's adolescents live. Most of the reports were of very small studies, with 8 of the 11 studies including fewer than 50 participants. The three randomised controlled trials were all small, with 34 26, 37 18 and 65 19 participants, respectively. The only two studies with over 100 participants were based on a retrospective cohort study in USA with a total of 174 participants over the three eras of care that were compared 20, and a very large multicountry study that used routinely reported clinic data 25.

The service delivery interventions that were identified in this review were predominantly focused on relatively resource‐intensive approaches applied at the individual level, such as motivational interviewing 26, counselling and peer support 19, 22, 24, 27, education 19, directly observed therapy 23, 28 and financial incentives 21. This likely reflects attempts to overcome the psychological, behavioural and educational difficulties experienced by adolescents (which may be contributory to poor outcomes) 29. However, interventions targeted at this level alone are unlikely to be generalisable or sufficient to overcome the considerable structural and health system barriers faced by adolescents. Complementary interventions, acting at different levels, are likely to be required. A recent systematic review of interventions to improve linkage from HIV diagnosis to initiation of ART amongst all HIV‐infected people in low‐ and middle‐income countries similarly identified a small number of studies, with most judged to be of poor quality, and the authors noted that few of the studies evaluated interventions specifically targeted to adolescents 30.

In contrast to the situation for adolescents, a greater number of interventions to improve adults' linkage, retention and adherence to ART have been evaluated and have been introduced into routine clinical practice, especially in resource‐limited settings 30, 31. At the policy and health systems levels, task‐shifting to lower cadres of health workers and non‐medical personal, decentralising care to primary care level and home delivery of HIV care including ART initiation has increased retention in care and improved acceptability 32, whilst decentralising care to primary care level has improved accessibility 33. Home initiation and delivery of HIV care have been shown to be effective in improving linkage and retention in care in well‐powered cluster‐randomised controlled trials 34, 35. Integration of HIV care services into general medical clinics has been widely implemented 36. At first glance, such interventions seem to be promising approaches that could be offered to adolescents. However, adolescents may have substantially different clinical, social and emotional needs than adults and unmodified implementation of interventions that have been designed for adults may not be appropriate. One example of an adolescent‐tailored intervention identified in this review was ‘adolescent‐friendly’ clinics, with special opening hours for adolescents and targeted services 25. However, limitations in the study's application of interventions and lack of clear evidence of effectiveness preclude recommendation of this strategy until further high‐quality studies are conducted.

This study had important strengths, including the comprehensive search strategy, rigorous methods and wide scope covering all aspects of the HIV care pathway for adolescents who had been diagnosed with HIV. However, there were limitations. Of principal concern was the apparent low‐to‐moderate quality of the majority of studies, meaning that any policy recommendations for implementation must be made with extreme caution until further, high‐quality studies have been conducted and adequately reported. Age definitions of adolescence varied widely between studies, and some studies that included adolescent participants were excluded because the majority of participants were not aged between 10 and 19 years, or we were unable to extract data for this age group 37, 38, 39, 40, 41, 42, 43, 44, 45, 46, 47, 48, 49, 50, 51, 52, 53, 54, 55, 56, 57, 58, 59, 60, 61, 62, 63, 64, 65, 66, 67, 68, 69, 70, 71, 72, 73, 74, 75, 76. In informal review of these studies, most of the interventions assessed were broadly similar to those included in the review and also had low methodological and/or reporting quality.

Because of the wide scope of the study and large numbers of possible study types included, it is possible that a small number of studies were inadvertently excluded because of our search or inclusion criteria. For example, due to time and logistic constraints, we only included studies published in English. This may partly explain the lack of studies from Latin America. In accordance with our published study protocol, we did not attempt to estimate summary measures of effectiveness for interventions due to the high degree of heterogeneity in the interventions identified.

## Conclusions and recommendations

Over a period of 13.5 years, only 11 studies examining the effect of interventions on adolescents' linkage, retention or adherence to ART were identified, with most studies conducted in high‐income countries and using relatively resource‐intensive, individual client approaches. Overall, the methodological quality of the reports of the identified studies was poor, and most studies had very few participants. Although qualified by the poor quality of studies, some approaches appear promising and need to be evaluated in larger, rigorous studies. These approaches, along with those identified in studies that had a combination of adults and adolescents 30, 32, 33, 77, 78, are summarised in Box 1.

Box 1 **Interventions identified in this review that warrant further investigation**

- For all HIV infected adolescents:
  ○ Improved accessibility to clinics and availability of youth‐friendly services
  ○ Multidisciplinary adolescent HIV clinics
  ○ Peer counselling and support
- For adolescents experiencing treatment failure or poor adherence:
  ○ Counselling (individual and family)
  ○ Financial incentives
  ○ Provision of adherence support devices (e.g. pill boxes and beepers)

Adolescents living with HIV are currently a neglected group. To ensure they are able to achieve the benefits of ART, rigorous evaluation of a wider range of existing and innovative service delivery interventions applied at the individual, health facility and policy levels are urgently required. When designing interventions, studies need to be cognisant of the limited resources available within health systems, which are particularly constrained in the countries with generalised HIV epidemics and large numbers of HIV‐infected adolescents.

## Funding

This work was commissioned by the World Health Organization. The content is solely the responsibility of the authors and does not necessarily represent the official views of the World Health Organization.

## Supporting information


**Figure S1.** Search strategy.
**Figure S2.** PRISMA flow diagram.
**Table S1.** (a) Characteristics of studies reporting on interventions to improve adolescents' *linkage* to ART. (b) Characteristics of studies reporting on interventions to improve adolescents' *retention* on ART. (c) Characteristics of studies reporting on interventions to improve adolescents' *adherence* to ART.
**Table S2.** Characteristics of excluded studies.
**Table S3**. Detailed risk of bias assessment for included studies.
