# Supplementary material for: Service delivery interventions to improve adolescents' linkage, retention and adherence to antiretroviral therapy and HIV care
Source: Trop Med Int Health. 2015 May 13;20(8):1015–32. doi: 10.1111/tmi.12517 (PMC4579546; doi:10.1111/tmi.12517)
Supplement: Supplementary file 1 — Figure S1. Search strategy. Figure S2. PRISMA flow diagram. Table S1. (a) Characteristics of studies reporting on interventions to improve adolescents' linkage to ART. (b) Characteristics of studies reporting on interventions to improve adolescents' retention on ART. (c) Characteristics of studies reporting on interventions to improve adolescents' adherence to ART. Table S2. Characteristics of excluded studies. Table S3. Detailed risk of bias assessment for included studies. [file tmi0020-1015-sd1.docx]

# Supplementary Figure 1. Search strategy

| **Medline (via PubMed)** | **Scopus** | **Web of Science** |
| --- | --- | --- |
| 1. hiv[MeSH Terms] OR hiv[tw] | 1. TITLE-ABS-KEY(“hiv”) OR TITLE-ABS-KEY(“HIV”) OR TITLE-ABS-KEY(“human immunodeficiency virus”) OR TITLE-ABS-KEY(“human immune-deficiency virus”) | 1. (“hiv”) OR (“HIV”) OR (“human immunodeficiency virus”) OR (“human immune-deficiency virus”) |
| 1. (antiretroviral therapy[tw] OR anti-retroviral agents/therapeutic use[Mesh Terms] OR anti HIV agents[MeSH Terms] OR antiretroviral therapy, highly active[MeSH Terms] OR ART[tw] OR ARV*[tw] OR antiretroviral*[tw] OR anti-retroviral[tw] OR HAART[tw] OR cART[tw]) | 1. TITLE-ABS-KEY(“antiretroviral therapy”) OR TITLE-ABS-KEY(“anti-retroviral”) OR TITLE-ABS-KEY(“ART”) TITLE-ABS-KEY(“ARV*”) OR TITLE-ABS-KEY(“HAART”) OR TITLE-ABS-KEY(“cART”) | 1. (“antiretroviral therapy”) OR (“anti-retroviral”) OR (“ART”) OR (“ARV*”) OR (“HAART”) OR (“cART”) |
| 1. (pre-treatment[tw] OR pretreatment[tw] OR prior to treatment[tw] OR prior-to-treatment[tw] OR pre-ART[tw]) | 1. TITLE-ABS-KEY(“pre-treatment”) OR TITLE-ABS-KEY(“pretreatment”) OR TITLE-ABS-KEY(“prior to treatment”) OR TITLE-ABS-KEY(“prior-to-treatment”) OR TITLE-ABS-KEY(“pre-ART”) | 1. (“pre-treatment”) OR (“pretreatment”) OR (“prior to treatment”) OR (“prior-to-treatment”) OR (“pre-ART”) |
| 1. (loss to follow-up[tw] OR loss to follow up[tw] OR lost to follow-up[tw] OR lost to follow up[tw] OR loss-to-follow-up[tw] OR lost-to-follow-up[tw] OR loss to retention[tw] OR lost to retention[tw] OR treatment initiation[tw] OR retention OR retain* OR attrition) | 1. TITLE-ABS-KEY(“loss to follow-up”) OR TITLE-ABS-KEY(“loss to follow up”) OR TITLE-ABS-KEY(“lost to follow-up”) OR TITLE-ABS-KEY(“lost to follow up”) OR TITLE-ABS-KEY(“loss-to-follow-up”) OR TITLE-ABS-KEY(“lost-to-follow-up”) OR TITLE-ABS-KEY(“loss to retention”) OR TITLE-ABS-KEY(“lost to retention”) OR TITLE-ABS-KEY(“treatment initiation”) OR TITLE-ABS-KEY(“retention”) OR TITLE-ABS-KEY(“retain*”) OR TITLE-ABS-KEY(“attrition”) | 1. (“loss to follow-up”) OR (“loss to follow up”) OR (“lost to follow-up”) OR (“lost to follow up”) OR (“loss-to-follow-up”) OR (“lost-to-follow-up”) OR (“loss to retention”) OR (“lost to retention”) OR (“treatment initiation”) OR (“retention”) OR (“retain*”) OR (“attrition”) |
| 1. (link*[tw] OR link to care[tw] OR link to treatment[tw] OR linkage to care[tw] OR linkage to treatment[tw] OR link into care[tw] OR linkage into care[tw] OR linkage into care[tw] OR linkage into treatment[tw]) | 1. TITLE-ABS-KEY(“link*”) OR TITLE-ABS-KEY(“link to care”) OR TITLE-ABS-KEY(“link to treatment”) OR TITLE-ABS-KEY(“linkage to care”) OR TITLE-ABS-KEY(“linkage to treatment”) OR TITLE-ABS-KEY(“link into care”) OR TITLE-ABS-KEY(“linkage into care”) OR TITLE-ABS-KEY(“linkage into care”) OR TITLE-ABS-KEY(“linkage into treatment”) | 1. (“link*”) OR (“link to care”) OR (“link to treatment”) OR (“linkage to care”) OR (“linkage to treatment”) OR (“link into care”) OR (“linkage into care”) OR (“linkage into care”) OR (“linkage into treatment”) |
| 1. (eligibility[tw] OR eligible[tw]OR eligib*) | 1. TITLE-ABS-KEY(“eligibility”) OR TITLE-ABS-KEY(“eligible”) OR TITLE-ABS-KEY(“eligib*”) | 1. (“eligibility”) OR (“eligible”) OR (“eligib*”) |
| 1. (Medication Adherence[MeSH Terms] OR Patient Compliance[MeSH Terms OR adher*[tw] OR complian*[tw] OR comply[tw] OR complied[tw] OR noncomplian*[tw] OR non-complian*[tw] OR non-adher*[tw] OR nonadher*[tw]) | 1. TITLE-ABS-KEY(“adher*”) OR TITLE-ABS-KEY(“complian*”) OR TITLE-ABS-KEY(“comply”) OR TITLE-ABS-KEY(“complied”) OR TITLE-ABS-KEY(“noncomplian*”) OR TITLE-ABS-KEY(“non-complian*”) OR TITLE-ABS-KEY(“non-adher*”) OR TITLE-ABS-KEY(“nonadher”) | 1. (“adher*”) OR (“complian*”) OR (“comply”) OR (“complied”) OR (“noncomplian*”) OR (“non-complian*”) OR (“non-adher*”) OR (“nonadher”) |
| 1. (Adolescent[MeSH Terms] OR youth[tw] OR young[tw] OR pediatri*[tw] OR paediatric*[tw] OR teenag*[tw] OR child*[tw] OR adolesc*[tw] OR adoles*[tw] OR child*[tw] OR young adult*[tw]) | 1. TITLE-ABS-KEY(“youth”) OR TITLE-ABS-KEY(“young”) OR TITLE-ABS-KEY(“pediatri*”) OR TITLE-ABS-KEY(“paediatric*”) OR TITLE-ABS-KEY(“teenag*”) OR TITLE-ABS-KEY(“child*”) OR TITLE-ABS-KEY(“adolesc*”) OR TITLE-ABS-KEY(“adoles*”) OR TITLE-ABS-KEY(“child*”) OR TITLE-ABS-KEY(“young adult*”) | 1. (“youth”) OR (“young”) OR (“pediatri*”) OR (“paediatric*”) OR (“teenag*”) OR (“child*”) OR (“adolesc*”) OR (“adoles*”) OR (“child*”) OR (“young adult*”) |
| 1. (#1 AND #2) | 1. (#1 AND #2) | 1. (#1 AND #2) |
| 1. (#3 OR #4 OR #5 OR #6 OR #7) | 1. (#3 OR #4 OR #5 OR #6 OR #7) | 1. (#3 OR #4 OR #5 OR #6 OR #7) |
| 1. (#8 AND #9 AND #10) | 1. (#8 AND #9 AND #10) | 1. (#8 AND #9 AND #10) |
| 1. Limit 11: 2001/01/01 - current | 1. Limit 11: 2001/01/01 - current | 1. Limit 11: 2001/01/01 - current |

# Supplementary Figure 2. PRISMA flow diagram

**Databases**

**3434** Abstracts identified

**11** Included in qualitative synthesis

**63** Excluded

**5**  Not relevant

**41** Not adolescents^†^

**2** Ineligible study design

**7** Review, commentary, editorial, economic or mathematical modelling

**8** Other reasons

**74** Full text reviewed

**296** Duplicates excluded

**3138** Abstracts reviewed

**3070** Excluded

**2943**  Not relevant

**46** Not adolescents

**16** Reports on clinical treatment options

**65** Ineligible study design

**4** Identified from bibliography review

**2** Identified from conference abstracts

† Studies where the majority of participants were not adolescents (aged 10 to 19 years) or where the results were not disaggregated so it was not possible to examine results of subgroups where the majority were adolescents

| Supplementary Table 1a. Characteristics of studies reporting on interventions to improve adolescents’ *linkage* to ART | | | | | | | | | | |
| --- | --- | --- | --- | --- | --- | --- | --- | --- | --- | --- |
| **First Author** | **Year published** | **Year study conducted** | **Country** | **Setting** | **Study population** | **Age range of eligible participants**  **(Mean age, median age or % aged 10-19 years age of participants)** | **Number of participants included in study** | **Proportion male** | **Study design** | **Service delivery intervention assessed** |
| Lamb[^25^](#_ENREF_25) ^†^  † Included for both retention on ART and for linkage to ART | 2014 | 2005-2010 | Kenya, Mozambique, Rwanda, Tanzania | 160 HIV care clinics | All HIV care clinic attenders age 10 years or older enrolled at clinic | 10 years or older (subgroup analysis of 10-14 years old and 15-24 years old)  10 years or older: 312,335  10-14 years: 3794  15-24 years: 53,244 | 10 years or older: 312,335  10-14 years: 3794  15-24 years: 53,244 | 10 years or older: 33%  10-14 years: NR  15-24 years: 15% | Report of routinely collected programme data | 1. Clinic availability of adolescent-targeted services: dedicated clinic hours for adolescents, adolescent peer educators, and adolescent support groups  2. Clinic availability of youth-targeted services: screening for sexually transmitted infections, condoms and hormonal contraceptives, and education on high-risk substance abuse behaviour |

| Supplementary Table 1b. Characteristics of studies reporting on interventions to improve adolescents’ *retention* on ART | | | | | | | | | | |
| --- | --- | --- | --- | --- | --- | --- | --- | --- | --- | --- |
| **First Author** | **Year published** | **Year study conducted** | **Country** | **Setting** | **Study population** | **Age range of eligible participants**  **(Mean age, median age or % aged 10-19 years age of participants)** | **Number of participants included in study** | **Proportion male** | **Study design** | **Service delivery intervention assessed** |
| ***Studies including HIV-infected adolescents attending clinics, who had previous problems with retention or adherence, or poor treatment outcomes*** | | | | | | | | | | |
| No studies | | | | | | | | | | |
| ***Studies including all HIV-infected adolescents attending clinics*** | | | | | | | | | | |
| Davila[^20^](#_ENREF_20) | 2013 | 2002-2008 | USA | 1 clinic for uninsured HIV-infected individuals in Houston, Texas | HIV-positive African-American and Hispanic adolescents enrolled at clinic | 13-23 years old  (56.3% aged <20 years old) | 174 | 60% | Retrospective cohort study | 3 eras of care: decentralised care with no youth-specific programmes; centralised care with youth multidisciplinary clinics; centralised care with enhanced youth support activities |
| Lamb[^25^](#_ENREF_25) † | 2014 | 2005-2010 | Kenya, Mozambique, Rwanda, Tanzania | 160 HIV care clinics | All HIV care clinic attenders aged 10 years or older enrolled at clinic | 10 years or older (subgroup analysis of 10-14 years old and 15-24 years old)  (10-14 years: 3794  15-24 years: 53,244  10 years or older: 312,335) | 10-14 years: 3794  15-24 years: 53,244  10 years or older: 312,335 | 10 years or older: 33%  15-24 years: 15% | Report of routinely collected programme data | 1. Clinic availability of adolescent-targeted services: dedicated clinic hours for adolescents, adolescent peer educators, and adolescent support groups  2. Clinic availability of youth-targeted services: screening for sexually transmitted infections, condoms and hormonal contraceptives, and education on high-risk substance abuse |

† Included for both retention on ART and for linkage to ART

| Supplementary Table 1c. Characteristics of studies reporting on interventions to improve adolescents’ *adherence* to ART | | | | | | | | | | |
| --- | --- | --- | --- | --- | --- | --- | --- | --- | --- | --- |
| **First Author** | **Year published** | **Year study conducted** | **Country** | **Setting** | **Study population** | **Age range of eligible participants (Mean age, median age or % aged 10-19 years age of participants)** | **Number of participants included in study** | **Proportion male** | **Study design** | **Service delivery intervention assessed** |
| ***Studies including HIV-infected adolescents attending clinics, who had previous problems with retention or adherence, or poor treatment outcomes*** | | | | | | | | | | |
| Foster[^21^](#_ENREF_21) | 2014 | 2010 | UK | A young persons HIV clinic | All patients with perinatally-acquired HIV who had transitioned from paediatric services to a specialist young persons HIV clinic with severe immunosuppression (CD4 count <200 cells/lL), currently off ART despite multiple attempts to restart, and were willing to restart therapy | 16-25 years old  (Median=19 years) | 12 | 25% | Prospective cohort study | Financial incentives linked to HIV viral load results, and multidisciplinary adherence support |
| Glikman[^23^](#_ENREF_23) | 2007 | 2004-2006 | USA | I hospital in Chicago | HIV care clinic attenders with virological failure on ART, no response to previous intensified support and care-giver report of perfect adherence | 7-17 years old  (Median=13 years) | 9 | 22% | Retrospective cohort study | Inpatient DOT for 7 days, supported by education from physicians, nurses, nutrition specialists, and social workers |
| Kaihin[^24^](#_ENREF_24) | 2014 | 2011 | Thailand | Two hospitals in Roi Et Province, North Eastern Thailand | HIV-positive youth taking ART at HIV care clinics and with adherence <95% | 15-24 years old  (Mean age: 18.2 years [intervention], 19.3 years [control]) | 46 | 43% | Prospective cohort study | Weekly nurse-led group sessions for 18 weeks aimed at increasing empowerment |
| Letourneau[^26^](#_ENREF_26) | 2013 | NR | USA | Two paediatric clinics (not further described) | HIV-positive patients receiving ART with either self-reported adherence<80%, or at least one HIV viral load >10,000 copies/ml, or physician decision to stop ART due to poor adherence | 9-17 years old  (Mean=15 years) | 34 | 35% | Randomised controlled trial | “Multisystemic therapy”, consisting of: (1) identification of risk factors across youths’ ecological systems; (2) use of individualized interventions that integrate empirically based clinical treatments into a broader ecological framework; (3) focusing interventions on caregivers to more effectively parent and on adolescents to more effectively cope (4) home- based treatment delivery (intervention group)  Or  Usual care with motivational interviewing, and financial incentives for attendance (control group) |
| Lyon[^27^](#_ENREF_27) | 2003 | 1998-2000 | USA | An urban children’s hospital (not further described) | HIV clinic attenders identified as having adherence difficulties by case managers. Most immunosuppressed were preferentially selected to participate | 15-23 years old  (Mean age=19.3 years) | 23 | 35% | Report of routinely collected programme data | 12-week multidisciplinary family group meetings for delivery of HIV educational curriculum and peer support |
| Parsons[^28^](#_ENREF_28) | 2006 | 2000-2003 | USA | 1 HIV specialist care clinic and rehabilitation facility in Baltimore | All HIV-positive patients receiving ART who were hospitalised because of concerns about adherence at a rehabilitation facility | 0.8-16 years  (Mean=11.1 years) | 23 (19 analysed) | 53% | Report of routinely collected programme data | DOT provided as part of multidisciplinary inpatient care. |

| **Supplementary Table 1c.** Characteristics of studies reporting on interventions to improve adolescents’ *adherence* to ART – cont’d | | | | | | | | | | | |
| --- | --- | --- | --- | --- | --- | --- | --- | --- | --- | --- | --- |
| **First Author** | **Year published** | **Year study conducted** | **Country** | **Setting** | **Study population** | **Age range of eligible participants**  **(Mean age, median age or % aged 10-19 years age of participants)** | **Number of participants included in study** | **Proportion male** | | **Study design** | **Service delivery intervention assessed** |
| ***Studies including all HIV-infected adolescents attending clinics*** | | | | | | | | | | | |
| Berrian[^18^](#_ENREF_18) | 2004 | 2000-2001 | USA | 1 HIV care referral centre in Connecticut | HIV infected children and youth attending clinic | 20 years old or younger  (Mean=10 years) | 37 | Intervention group: 45%  Control group: 55% | Randomised controlled trial | | Eight structured home visits by a single nurse over three months. Sessions included counselling, education, development of individualized care plan and provision of adherence reminders (e.g. medication boxes and beepers (intervention group).  Or  Clinic-based standard of care only (control group) |
| Bhana[^19^](#_ENREF_19) | 2014 | NR | South Africa | Two hospitals in Kwa-Zulu Natal | HIV-positive patients enrolled at HIV care clinics | 10-14 years old  (100% aged between 10 and 13 years old) | 65 | 49% | Randomised controlled trial | | Multi-session (6 over 3 months), family-based counselling with cartoon aids |
| Funck-Brentano[^22^](#_ENREF_22) | 2005 | 1999-2003 | France | 1 hospital in Paris | Patients infected perinatally and attending HIV care clinic | 12-17 years old  (Median=14.2 years) | 30 | 37% | Prospective cohort study | | 90-min group therapy and peer support session once every 6 weeks for 26 months |

NR: Not reported, ART: antiretroviral therapy, DOT: directly observed therapy

| **Supplementary Table 2: Characteristics of excluded studies** | | |
| --- | --- | --- |
|  | STUDY | REASON FOR EXCLUSION |
| 1 | Adjorlolo-Johnson 2013[^1^](#_ENREF_1) | Not relevant: study of associations between clinic child volume and clinic characteristics |
| 2 | Altice 2010[^2^](#_ENREF_2) | Comment |
| 3 | Bain-Brickley 2011[^3^](#_ENREF_3) | Systematic review |
| 4 | Basso 2013[^4^](#_ENREF_4) | Majority of participants >19 years, and not possible to extract data for all or part of 10-19 year age group (eligible: >17 years, mean age=42 years) |
| 5 | Belzer[^5^](#_ENREF_5) | Majority of participants >19 years, and not possible to extract data for all or part of 10-19 year age group (eligible: 15-24 years, mean age=20 years) |
| 6 | Braitstein 2012[^6^](#_ENREF_6) | Majority of participants >19 years, and not possible to extract data for all or part of 10-19 year age group (eligible >13 years old, median age=36 years) |
| 7 | Chandwani 2011[^7^](#_ENREF_7) | Not relevant: effect of interventions on linkage, retention or adherence not assessed |
| 8 | Chung 2005[^8^](#_ENREF_8) | Participants stated to be “adults”, age range not given |
| 9 | de Bruin 2010[^9^](#_ENREF_9) | Majority of participants >19 years, and not possible to extract data for all or part of 10-19 year age group (eligible: 18 years or older, mean age=48 years) |
| 10 | Dieckhaus 2007[^10^](#_ENREF_10) | Majority of participants >19 years, and not possible to extract data for all or part of 10-19 year age group (age range of participants: 12-73 years, mean age: 43 years). |
| 11 | Dilorio 2003[^11^](#_ENREF_11) | Adults (age range: 26-53 years) |
| 12 | do Carmo Sales Monteiro 2008[^12^](#_ENREF_12) | No retention, linkage or adherence outcome data reported |
| 13 | Dowashen 2011[^13^](#_ENREF_13) | Majority of participants >19 years, and not possible to extract data for all or part of 10-19 year age group (eligible: 12-29 years, mean age 23 years) |
| 14 | Dowashen 2012[^14^](#_ENREF_14) | Majority of participants >19 years, and not possible to extract data for all or part of 10-19 year age group (eligible: 14-29 years, mean age 23 years) |
| 15 | Dowashen 2013[^15^](#_ENREF_15) | Majority of participants <10 years or >19 years, and not possible to extract data for all or part of 10-19 year age group (eligible: 14-29 years, mean age 23 years) |
| 16 | Fatti 2014[^16^](#_ENREF_16) | Children (median age: 6 years) |
| 17 | Finocchario-Kessler 2012[^17^](#_ENREF_17) | Adults (mean age: 40 years) |
| 18 | Gardner 2005[^18^](#_ENREF_18) | Majority of participants >19 years, and not possible to extract data for all or part of 10-19 year age group (eligible: 18 years or older, 11% aged 18-25 years old). |
| 19 | Garvie 2007[^19^](#_ENREF_19) | Not possible to extract data for all or part of 10-19 year age group (participants aged between 4 and 21 years old, but no age distribution or age-disaggregated data presented) |
| 20 | Gaur 2010[^20^](#_ENREF_20) | Majority of participants >19 years, and not possible to extract data for all or part of 10-19 year age group (eligible: 16-24 years old, mean age: 21 years) |
| 21 | Giordano 2013[^21^](#_ENREF_21) | Adults (mean age 44 years old) |
| 22 | Goggin 2013[^22^](#_ENREF_22) | Majority of participants >19 years, and not possible to extract data for all or part of 10-19 year age group (eligible: 18 years or older, mean age=40 years) |
| 23 | Haberer 2013[^23^](#_ENREF_23) | Majority of participants <10 years or >19 years, and not possible to extract data for all or part of 10-19 year age group (described as “adults”, mean age=37 years; and “children”, mean age=7 years) |
| 24 | Hailey 2013[^24^](#_ENREF_24) | No relevant outcomes presented |
| 25 | Halperin 2013[^25^](#_ENREF_25) | Adults (mean age: 40 years) |
| 26 | Harris 2003[^26^](#_ENREF_26) | Rates of retention, or effect of interventions on retention in pre-ART care for subset of HIV-positive participants (37/1426, 2.6%) not reported |
| 27 | Hightow-Weidman 2011[^27^](#_ENREF_27) | Not relevant: baseline characteristics of trial population reported, no retention, linkage or adherence outcome assessed |
| 28 | Hightow-Weidman 2011[^28^](#_ENREF_28) | Majority of participants >19 years, and not possible to extract data for all or part of 10-19 year age group (mean age: 21 years) |
| 29 | Horvath 2012[^29^](#_ENREF_29) | Systematic review |
| 30 | Igumbor 2011[^30^](#_ENREF_30) | Majority of participants >19 years, and not possible to extract data for all or part of 10-19 year age group (all ages of participants eligible, 59% aged between 25 and 39 years old) |
| 31 | Jani 2011[^31^](#_ENREF_31) | Majority of participants <10 years or >19 years, and not possible to extract data for all or part of 10-19 year age group (eligible: older than 1 year old, relevant age disaggregated data not reported) |
| 32 | Kamau 2012[^32^](#_ENREF_32) | Majority of participants >19 years, and not possible to extract data for all or part of 10-19 year age group (eligible: 18 years or older, 21% aged between 18 and 30 years old) |
| 33 | Kenya 2013[^33^](#_ENREF_33) | Majority of participants >19 years, and not possible to extract data for all or part of 10-19 year age group (eligible: 18 years or older, 4% aged between 18 and 24 years old) |
| 34 | Kunutsor 2010[^34^](#_ENREF_34) | Majority of participants >19 years, and not possible to extract data for all or part of 10-19 year age group (eligible: 18 years and older, mean age=38.2 years) |
| 35 | Kunutsor 2012[^35^](#_ENREF_35) | Majority of participants >19 years, and not possible to extract data for all or part of 10-19 year age group (eligible: 18 years or older, 5% aged 18-25 years old) |
| 36 | Lyon 2011[^36^](#_ENREF_36) | Not relevant: no intervention aimed at improving retention, linkage or adherence described |
| 37 | Magnano San Lio 2009[^37^](#_ENREF_37) | Majority of participants >19 years, and not possible to extract data for all or part of 10-19 year age group (eligible: older than 15 years, mean age 36 years old) |
| 38 | Magnus 2010[^38^](#_ENREF_38) | Majority of participants >19 years, and not possible to extract data for all or part of 10-19 year age group (eligible: between 13 and 24 years old, 19% <19 years old) |
| 39 | Musiime 2007[^39^](#_ENREF_39) | Conference abstract. Insufficient data reported to allow assessment of effect of interventions |
| 40 | Naar-King 2009[^40^](#_ENREF_40) | Majority of participants >19 years, and not possible to extract data for all or part of 10-19 year age group (eligible: 16-24 years old, mean age=20 years old) |
| 41 | Naar-King 2013[^41^](#_ENREF_41) | Majority of participants >19 years, and not possible to extract data for all or part of 10-19 year age group (eligible 16-24 years old, mean age: 20 years old) |
| 42 | Outlaw 2014[^42^](#_ENREF_42) | Feasibility study – no retention, linkage or adherence outcome investigated |
| 43 | Palmer 2004[^43^](#_ENREF_43) | Review |
| 44 | Patten 2013[^44^](#_ENREF_44) | Majority of participants >19 years, and not possible to extract data for all or part of 10-19 year age group (eligible: 12-25 years old, median age: 22 years) |
| 45 | Puccio 2006[^45^](#_ENREF_45) | Majority of participants >19 years, and not possible to extract data for all or part of 10-19 year age group (eligible: 16-24 years old, 12% <20 years old) |
| 46 | Purdy 2008[^46^](#_ENREF_46) | Case series of 5 selected patients with complex treatment histories receiving directly observed therapy |
| 47 | Rawlings 2003[^47^](#_ENREF_47) | Majority of participants >19 years, and not possible to extract data for all or part of 10-19 year age group (eligible: 18 years or older, mean age 37 years old) |
| 48 | Reisner 2009[^48^](#_ENREF_48) | Review |
| 49 | Reynolds 2001[^49^](#_ENREF_49) | Not possible to extract data for all or part of 10-19 year age group (eligible 1-19 years old, no age distribution or age disaggregated data reported) |
| 50 | Rogers 2001[^50^](#_ENREF_50) | Effect of interventions on linkage, retention or adherence not assessed |
| 51 | Rongkavilit 2013[^51^](#_ENREF_51) | Majority of participants >19 years, and not possible to extract data for all or part of 10-19 year age group (eligible 16-25 years old, mean age=22 years old) |
| 52 | Rongkavilit 2014[^52^](#_ENREF_52) | Not relevant: effect of interventions on linkage, retention or adherence not assessed |
| 53 | Rotheram-Borus 2004[^53^](#_ENREF_53) | Majority of participants >19 years, and not possible to extract data for all or part of 10-19 year age group (eligible 16-29 years old, median age 23 years) |
| 54 | Rueda 2006[^54^](#_ENREF_54) | Systematic review |
| 55 | Saberi 2013[^55^](#_ENREF_55) | Qualitative study |
| 56 | Saberi 2014[^56^](#_ENREF_56) | Majority of participants >19 years, and not possible to extract data for all or part of 10-19 year age group (eligible 12-24 years old, mean age=20 years) |
| 57 | Shegog 2012[^57^](#_ENREF_57) | Feasibility study – no retention, linkage or adherence outcome investigated |
| 58 | Simoni 2009[^58^](#_ENREF_58) | Majority of participants >19 years, and not possible to extract data for all or part of 10-19 year age group (eligible: 18 years or older, mean age=40 years) |
| 59 | Snyder 2014[^59^](#_ENREF_59) | Majority of participants >19 years, and not possible to extract data for all or part of 10-19 year age group (eligible: 16-24 years old, median=22 years) |
| 60 | Van Der Plas 2013[^60^](#_ENREF_60) | Not relevant: effect of interventions on linkage, retention or adherence not assessed |
| 61 | Van Winghem 2008[^61^](#_ENREF_61) | Majority of participants <10 years and not possible to extract data for all or part of 10-19 year age group (eligible: <15 years old, median age 4.8 years) |
| 62 | Wohl 2011[^62^](#_ENREF_62) | Majority of participants >19 years, and not possible to extract data for all or part of 10-19 year age group (eligible 18-24 years old, mean age: 21 years) |
| 63 | Wynberg 2014[^63^](#_ENREF_63) | Systematic review |

# Supplementary Table 3: Detailed risk of bias assessment for included studies

| **Berrian 2004**[**^64^**](#_ENREF_64) |  |  |
| --- | --- | --- |
| **Bias** | **Author’s judgement** | **Support for judgement** |
| Random sequence generation | Low risk | Random number table used |
| Allocation concealment | Unclear | Procedures for allocation not clearly described |
| Blinding of participants and personnel | High risk | Study nurses provided intervention and supported participants to complete adherence questionnaire |
| Incomplete outcome data | Low risk | Outcome data available for most participants, and missing data accounted for |
| Selective reporting | Unclear | States that multivariate modelling undertaken to adjust for differences between groups in baseline characteristics (e.g. sex, disease stage), but not clearly reported |
| Other bias | High risk | Small numbers, only 33% of those invited to participate did so. Non validated adherence score |

| **Bhana 2014**[**^65^**](#_ENREF_65) |  |  |
| --- | --- | --- |
| **Bias** | **Author’s judgement** | **Support for judgement** |
| Random sequence generation | Unclear | Randomisation procedures not described |
| Allocation concealment | Unclear | Allocation procedures not described |
| Blinding of participants and personnel | Unclear | Not described |
| Incomplete outcome data | High risk | Only 55% attended all 6 sessions. Only summary measures presented for outcomes |
| Selective reporting | High risk | No measure of relative or absolute effect of intervention or confidence intervals presented |
| Other bias | High risk | Insufficient details of procedures for ascertainment of outcomes. Adherence measure (*“last time missed meds”*) not clearly defined. No description of handling of missing data. |

| **Letourneau 2013**[**^66^**](#_ENREF_66) |  |  |
| --- | --- | --- |
| **Bias** | **Author’s judgement** | **Support for judgement** |
| Random sequence generation | High risk | Not random, but based on sequence of clinic attendance. |
| Allocation concealment | High risk | By nature of randomisation procedure, allocation not concealed |
| Blinding of participants and personnel | High risk | Not blinded. Adherence data collected by researchers during follow-up visits |
| Incomplete outcome data | High risk | Numbers randomised, allocated and excluded do not sum in flow diagram. Handling of missing outcome data not clearly reported |
| Selective reporting | High risk | Odds ratios, but not confidence intervals reported. Baseline characteristics of groups not reported. Analysis not adjusted for possible differences (e.g. baseline HIV viral load) between groups). |
| Other bias | High risk | Content of two interventions not clearly distinguished, some overlap In interventions between groups. Poor adherence defined to be <90%. |

**Non-randomised studies**

| **Davila 2013**[**^67^**](#_ENREF_67) |  |  |
| --- | --- | --- |
| **Bias** | **Author’s judgement** | **Support for judgement** |
| Selection to be representative of target population | Unclear | Recruited all HIV-infected African-American and Hispanic patients between the ages of 13 and 23 years who entered care at one clinic between 1 January 2002 and 31 August 2008 |
| Clear selection criteria, avoiding inappropriate exclusions | Low risk | All eligible participants attending clinic |
| Interventions applied consistently | High risk | Exposure was “era”, but not clear if all (or any) of participants categorized as exposed to era received interventions, or whether they could receive more than more intervention (e.g. if in service long enough). Some interventions appear to be available over more than one era. |
| Potential confounders identified and adjusted for | High risk | Effect of exposure likely to be influenced by secular trends. No adjustment for ART treatment regimens. Not adjusted for CD4 count or HIV viral load at baseline. Competing hazards (mortality, loss-to-follow-up, transfer) not addressed. |
| Ascertainment of outcome sufficient | Unclear | Handling of clinic attendance data not described. Specifically notes that: “*Information on missed appointments, whether a visit was as originally scheduled or rescheduled, and visits outside [the clinic] was not available.”* |
| Sufficient follow-up | Low risk | Retention assessed after 12-months follow-up |
| Incomplete outcome data | High risk | Missing data on clinic attendance not described – could be falsely equated with non-retention |
| Selective outcome reporting | High risk | Loss-to-follow-up, rates of transfer and mortality not reported |
| Other biases | High risk | Only one clinic. No external comparison group. Retrospective analysis, so no tracing to ascertain outcomes |

| **Foster 2014**[**^68^**](#_ENREF_68) |  |  |
| --- | --- | --- |
| **Bias** | **Author’s judgement** | **Support for judgement** |
| Selection to be representative of target population | Low risk | Representative of adolescents with treatment failure and who were willing to restart ART |
| Clear selection criteria, avoiding inappropriate exclusions | Low risk | No concerns |
| Interventions applied consistently | Low risk | Clear pre-defined protocol for provision of financial incentives |
| Potential confounders identified and adjusted for | High risk | Analysis not adjusted for effect of confounders (but small numbers recruited). |
| Ascertainment of outcome sufficient | Low risk | HIV viral load measured routinely at 12 and 24 weeks |
| Sufficient follow-up | Low risk | 24 months |
| Incomplete outcome data | Low risk | No missing data for outcome |
| Selective outcome reporting | Low risk | No evidence of selective reporting of outcomes |
| Other biases | High risk | Small study (n=11), no comparison group |

| **Funck-Brentano 2005**[**^69^**](#_ENREF_69) |  |  |
| --- | --- | --- |
| **Bias** | **Author’s judgement** | **Support for judgement** |
| Selection to be representative of target population | High risk | Participants selected on basis of: “*evidence or motivation to participate in peer support*”. |
| Clear selection criteria, avoiding inappropriate exclusions | High risk | Excluded participants who were assessed as not being motivated for participation |
| Interventions applied consistently | Low risk | Open-ended group discussions held regularly |
| Potential confounders identified and adjusted for | High risk | Comparisons between groups not adjusted for confounders |
| Ascertainment of outcome sufficient | High risk | States that patients’ adherence was measured “*as subjectively perceived by the pediatrician*.” |
| Sufficient follow-up | Low risk | 26 months follow-up |
| Incomplete outcome data | Unclear | Not described |
| Selective outcome reporting | High risk | Differences between groups in baseline characteristics not reported. Outcome not adjusted for possible differences (although numbers small). |
| Other biases | High risk | Small numbers. Fidelity to intervention not clearly described: “*attendance fluctuated*”. |

| **Glikman 2007**[**^70^**](#_ENREF_70) |  |  |
| --- | --- | --- |
| **Bias** | **Author’s judgement** | **Support for judgement** |
| Selection to be representative of target population | Low risk | Children selected for admission for DOT as had predefined criteria for treatment failure and adherence difficulties |
| Clear selection criteria, avoiding inappropriate exclusions | Low risk | Criteria for treatment failure clearly defined |
| Interventions applied consistently | Low risk | States that “*Under direct observation of the nursing staff, patients were administered exactly the same HAART regimen as prescribed at home. Timing of administration was similar to the home schedule of the patient.”* |
| Potential confounders identified and adjusted for | High risk | No adjustment for potential confounders (although numbers small) |
| Ascertainment of outcome sufficient | Low risk | HIV viral loads measured regularly according to protocol |
| Sufficient follow-up | Low risk | Participants followed-up for 6months |
| Incomplete outcome data | Low risk | No missing HIV viral load data |
| Selective outcome reporting | High risk | Figure 1 presents *“typical patterns of HIV VL detected over time”.* Not clear if data from actually participants or illustrative data. |
| Other biases | High risk | Small numbers of participants (n=9). No comparison group. |

| **Kaihin 2014**[**^71^**](#_ENREF_71) |  |  |
| --- | --- | --- |
| **Bias** | **Author’s judgement** | **Support for judgement** |
| Selection to be representative of target population | Unclear | States participants were randomly selected, but no methods for random selection provided. |
| Clear selection criteria, avoiding inappropriate exclusions | High risk | Rationale for selecting and allocating hospitals to intervention groups not provided. |
| Interventions applied consistently | Unclear | Insufficient details of fidelity to motivational interviewing intervention |
| Potential confounders identified and adjusted for | High risk | Baseline HIV viral load and CD4 count not adjusted for. |
| Ascertainment of outcome sufficient | High risk | Outcome assessed on the basis of a single pill count at session 8. High possibility of social desirability bias. States that pharmacists were blinded to interventions, but seems infeasible given study design |
| Sufficient follow-up | Low risk | No concerns |
| Incomplete outcome data | Low risk | Not explicitly stated, but from Table 2, assume no missing outcome data. |
| Selective outcome reporting | Low risk | No concerns |
| Other biases | High risk | High likelihood of Hawthorne effect. Only 2 clinics compared. No adjustment of measures of uncertainty for clustering. Small numbers of participants (23 per clinic) |

| **Lamb 2014**[**^72^**](#_ENREF_72) |  |  |
| --- | --- | --- |
| **Bias** | **Author’s judgement** | **Support for judgement** |
| Selection to be representative of target population | Low risk | Participants aged 10 or older from 160 clinics in 4 countries between 2005 and 2010 |
| Clear selection criteria, avoiding inappropriate exclusions | Low risk | No inappropriate exclusion |
| Interventions applied consistently | Unclear | Availability of clinic-level “adolescent targeted services” and “services likely to be used by youth” retrospectively coded by study employees. Unclear if interventions consistently available or applied across clinics |
| Potential confounders identified and adjusted for | Low risk | Appropriately adjusted models for pre-ART and ART attrition |
| Ascertainment of outcome sufficient | Low risk | Clear, standard definitions of attrition. Attrition ascertained through high-quality clinic-level data collection system |
| Sufficient follow-up | Low risk | For both pre-ART attrition and ART attrition, assessed at 1 yea following recruitment |
| Incomplete outcome data | Low risk | No concerns |
| Selective outcome reporting | Low risk | No concerns |
| Other biases | High risk | High proportion of participants missing CD4 count (15-24 years: 34%, >10 years: 32%). Low CD4 count known to be associated with risk of attrition. Clinic “Youth targeted services” are in fact applicable to most HIV infected individuals. |

| **Lyon 2003**[**^73^**](#_ENREF_73) |  |  |
| --- | --- | --- |
| **Bias** | **Author’s judgement** | **Support for judgement** |
| Selection to be representative of target population | High risk | 30/200 adolescents attending clinic purposefully selected by researchers to participate. Adolescents with lowest CD4 cell counts selected first |
| Clear selection criteria, avoiding inappropriate exclusions | High risk | Case managers identified adolescents with reported difficulties adhering to ART (not defined how ascertained). Groups meant to be closed after 10 members recruited, but in 3 consecutive groups, 8,7 and 8 participants were recruited. Exclusions not described. |
| Interventions applied consistently | Unclear | 5/23 didn’t complete sessions. Absences common during group sessions. |
| Potential confounders identified and adjusted for | High risk | Effect of participant characteristics and completion of sessions on outcomes not assessed or adjusted for (although numbers small). |
| Ascertainment of outcome sufficient | Low risk | No concerns |
| Sufficient follow-up | Low risk | Baseline and 12-week assessment |
| Incomplete outcome data | Low risk | 23/23 participants provided baseline and outcome data |
| Selective outcome reporting | Low risk | No concerns |
| Other biases | High risk | Small numbers, no control group. High possibility of Hawthorne effect with self-reported adherence |

| **Parsons 2006**[**^74^**](#_ENREF_74) |  |  |
| --- | --- | --- |
| **Bias** | **Author’s judgement** | **Support for judgement** |
| Selection to be representative of target population | Low risk | All participants hospitalised at one site for persistent virological failure despite intensive adherence support |
| Clear selection criteria, avoiding inappropriate exclusions | Low risk | No concerns |
| Interventions applied consistently | High risk | Described as “inpatient directly observed therapy”, but no description given of methods for observing therapy. Wide range of duration of exposure to interventions (4-22 days). |
| Potential confounders identified and adjusted for | High risk | Effect of participant characteristics on outcomes not assessed or adjusted for (although numbers small). |
| Ascertainment of outcome sufficient | High risk | Wide range of measurement of discharge viral load (4-22 days) |
| Sufficient follow-up | Low risk | No concerns |
| Incomplete outcome data | Low risk | No concerns |
| Selective outcome reporting | Low risk | No concerns |
| Other biases | High risk | Small numbers (n=19), no comparison group |

**References**

1. Adjorlolo-Johnson G, Wahl Uheling A, Ramachandran S, et al. Scaling up pediatric HIV care and treatment in Africa: clinical site characteristics associated with favorable service utilization. *Journal of acquired immune deficiency syndromes (1999)* 2013; **62**(1): e7-e13.

2. Altice FL, Springer SA. DAART for human immunodeficiency virus-infected patients: Studying subjects not at risk for nonadherence and use of untested interventions. *Archives of internal medicine* 2010; **170**(1): 109-10.

3. Bain-Brickley D, Butler LM, Kennedy GE, Rutherford GW. Interventions to improve adherence to antiretroviral therapy in children with HIV infection. *The Cochrane database of systematic reviews* 2011; (12): CD009513.

4. Basso CR, Helena ETS, Caraciolo JMM, Paiva V, Nemes MIB. Exploring ART intake scenes in a human rights-based intervention to improve adherence: A randomized controlled trial. *AIDS and Behavior* 2013; **17**(1): 181-92.

5. Belzer M. A pilot study using cell phone interactions to improve HIV medication adherence in adolescents who have previously failed antiretroviral therapy. *Journal of Adolescent Health* 2013; **52**(2): S7-S.

6. Braitstein P, Siika A, Hogan J, et al. A clinician-nurse model to reduce early mortality and increase clinic retention among high-risk HIV-infected patients initiating combination antiretroviral treatment. *Journal of the International AIDS Society* 2012; **15**(1): 7.

7. Chandwani S, Abramowitz S, Koenig LJ, Barnes W, D'Angelo L. A multimodal behavioral intervention to impact adherence and risk behavior among perinatally and behaviorally HIV-infected youth: description, delivery, and receptivity of adolescent impact. *AIDS education and prevention : official publication of the International Society for AIDS Education* 2011; **23**(3): 222-35.

8. Chung AM, Mancao N. A pharmacist-managed medication adherence program to improve antiretroviral therapy adherence and clinical outcomes in HIV/AIDS-infected adults and children. *Pharmacotherapy* 2005; **25**(3): 479-.

9. de Bruin M, Hospers HJ, van Breukelen GJ, Kok G, Koevoets WM, Prins JM. Electronic monitoring-based counseling to enhance adherence among HIV-infected patients: a randomized controlled trial. *Health psychology : official journal of the Division of Health Psychology, American Psychological Association* 2010; **29**(4): 421-8.

10. Dieckhaus KD, Odesina V. Outcomes of a multifaceted medication adherence intervention for HIV-positive patients. *AIDS patient care and STDs* 2007; **21**(2): 81-91.

11. DiIorio C, Resnicow K, McDonnell M, Soet J, McCarty F, Yeager K. Using motivational interviewing to promote adherence to antiretroviral medications: a pilot study. *The Journal of the Association of Nurses in AIDS Care : JANAC* 2003; **14**(2): 52-62.

12. do Carmo Sales Monteiro M, VanderVeken L, Zilhao I, et al. Increasing access to HIV/AIDS treatment and care for youth and adolescents in Mozambique. *Mexico City - AIDS 2008: Abstract no WEPE0561* 2008.

13. Dowshen N, Kuhns L, Johnson A, Holoyda B, Garofalo R. Text message reminders to improve adherence to antiretroviral therapy for HIV-positive youth. *Journal of Adolescent Health* 2011; **48**(2): S64-S5.

14. Dowshen N, Kuhns LM, Johnson A, Holoyda BJ, Garofalo R. Improving adherence to antiretroviral therapy for youth living with HIV/AIDS: a pilot study using personalized, interactive, daily text message reminders. *Journal of medical Internet research* 2012; **14**(2): e51.

15. Dowshen N, Kuhns LM, Gray C, Lee S, Garofalo R. Feasibility of interactive text message response (ITR) as a novel, real-time measure of adherence to antiretroviral therapy for HIV+ youth. *AIDS Behav* 2013; **17**(6): 2237-43.

16. Fatti G, Shaikh N, Eley B, Grimwood A. Improved virological suppression in children on antiretroviral treatment receiving community-based adherence support: a multicentre cohort study from South Africa. *AIDS care* 2014; **26**(4): 448-53.

17. Finocchario-Kessler S, Catley D, Thomson D, Bradley-Ewing A, Berkley-Patton J, Goggin K. Patient communication tools to enhance ART adherence counseling in low and high resource settings. *Patient education and counseling* 2012; **89**(1): 163-70.

18. Gardner LI, Metsch LR, Anderson-Mahoney P, et al. Efficacy of a brief case management intervention to link recently diagnosed HIV-infected persons to care. *AIDS (London, England)* 2005; **19**(4): 423-31.

19. Garvie PA, Lensing S, Rai SN. Efficacy of a pill-swallowing training intervention to improve antiretroviral medication adherence in pediatric patients with HIV/AIDS. *Pediatrics* 2007; **119**(4): e893-9.

20. Gaur AH, Belzer M, Britto P, et al. Directly observed therapy (DOT) for nonadherent HIV-infected youth: lessons learned, challenges ahead. *AIDS research and human retroviruses* 2010; **26**(9): 947-53.

21. Giordano TP, Rodriguez S, Zhang H, et al. Effect of a clinic-wide social marketing campaign to improve adherence to antiretroviral therapy for HIV infection. *AIDS Behav* 2013; **17**(1): 104-12.

22. Goggin K, Gerkovich MM, Williams KB, et al. A randomized controlled trial examining the efficacy of motivational counseling with observed therapy for antiretroviral therapy adherence. *AIDS Behav* 2013; **17**(6): 1992-2001.

23. Haberer JE, Kiwanuka J, Nansera D, et al. Realtime adherence monitoring of antiretroviral therapy among HIV-infected adults and children in rural Uganda. *AIDS (London, England)* 2013; **27**(13): 2166-8.

24. Hailey JH, Arscott J. Using technology to effectively engage adolescents and young adults into care: STAR TRACK Adherence Program. *The Journal of the Association of Nurses in AIDS Care : JANAC* 2013; **24**(6): 582-6.

25. Halperin J, Pathmanathan I, Richey LE. Disclosure of HIV status to social networks is strongly associated with increased retention among an urban cohort in New Orleans. *AIDS patient care and STDs* 2013; **27**(7): 375-7.

26. Harris SK, Samples CL, Keenan PM, Fox DJ, Melchiono MW, Woods ER. Outreach, mental health, and case management services: can they help to retain HIV-positive and at-risk youth and young adults in care? *Maternal and child health journal* 2003; **7**(4): 205-18.

27. Hightow-Weidman LB, Jones K, Phillips G, 2nd, Wohl A, Giordano TP. Baseline clinical characteristics, antiretroviral therapy use, and viral load suppression among HIV-positive young men of color who have sex with men. *AIDS patient care and STDs* 2011; **25 Suppl 1**: S9-14.

28. Hightow-Weidman LB, Smith JC, Valera E, Matthews DD, Lyons P. Keeping them in "STYLE": finding, linking, and retaining young HIV-positive black and Latino men who have sex with men in care. *AIDS patient care and STDs* 2011; **25**(1): 37-45.

29. Horvath T, Azman H, Kennedy GE, Rutherford GW. Mobile phone text messaging for promoting adherence to antiretroviral therapy in patients with HIV infection. *The Cochrane database of systematic reviews* 2012; **3**: CD009756.

30. Igumbor JO, Scheepers E, Ebrahim R, Jason A, Grimwood A. An evaluation of the impact of a community-based adherence support programme on ART outcomes in selected government HIV treatment sites in South Africa. *AIDS care* 2011; **23**(2): 231-6.

31. Jani IV, Sitoe NE, Alfai ER, et al. Effect of point-of-care CD4 cell count tests on retention of patients and rates of antiretroviral therapy initiation in primary health clinics: an observational cohort study. *Lancet* 2011; **378**(9802): 1572-9.

32. Kamau TM, Olsen VG, Zipp GP, Clark M. The effectiveness of social resource intervention to promote adherence to HIV medication in a multidisciplinary care setting in Kenya. *International journal of STD & AIDS* 2012; **23**(12): 843-8.

33. Kenya S, Jones J, Arheart K, et al. Using community health workers to improve clinical outcomes among people living with HIV: a randomized controlled trial. *AIDS Behav* 2013; **17**(9): 2927-34.

34. Kunutsor S, Walley J, Katabira E, et al. Using mobile phones to improve clinic attendance amongst an antiretroviral treatment cohort in rural Uganda: a cross-sectional and prospective study. *AIDS Behav* 2010; **14**(6): 1347-52.

35. Kunutsor S, Walley J, Muchuro S, et al. Improving adherence to antiretroviral therapy in sub-Saharan African HIV-positive populations: an enhanced adherence package. *AIDS care* 2012; **24**(10): 1308-15.

36. Lyon ME, Garvie PA, Kao E, et al. Spirituality in HIV-infected adolescents and their families: FAmily CEntered (FACE) advance care planning and medication adherence. *Journal of Adolescent Health* 2011; **48**(6): 633-6.

37. Magnano San Lio M, Mancinelli S, Palombi L, et al. The DREAM model's effectiveness in health promotion of AIDS patients in Africa. *Health Promotion International* 2009; **24**(1): 6-15.

38. Magnus M, Jones K, Phillips G, 2nd, et al. Characteristics associated with retention among African American and Latino adolescent HIV-positive men: results from the outreach, care, and prevention to engage HIV-seropositive young MSM of color special project of national significance initiative. *Journal of acquired immune deficiency syndromes (1999)* 2010; **53**(4): 529-36.

39. Musiime V, Kizito H, Ssali F, et al. An adolescent peer support group improves adherence to antiretroviral therapy and reduces self stigma among HIV infected adolescents at joint clinical research centre (JCRC), Kampala. *Sydney - IAS 2007: Abstract no CDB446* 2007.

40. Naar-King S, Parsons JT, Murphy DA, Chen X, Harris DR, Belzer ME. Improving health outcomes for youth living with the human immunodeficiency virus: a multisite randomized trial of a motivational intervention targeting multiple risk behaviors. *Archives of pediatrics & adolescent medicine* 2009; **163**(12): 1092-8.

41. Naar-King S, Outlaw AY, Sarr M, et al. Motivational Enhancement System for Adherence (MESA): pilot randomized trial of a brief computer-delivered prevention intervention for youth initiating antiretroviral treatment. *Journal of pediatric psychology* 2013; **38**(6): 638-48.

42. Outlaw AY, Naar-King S, Tanney M, et al. The initial feasibility of a computer-based motivational intervention for adherence for youth newly recommended to start antiretroviral treatment. *AIDS care* 2014; **26**(1): 130-5.

43. Palmer A. Motivating asymptomatic adolescents to adhere to HAART regimen can be challenging. *HIV clinician / Delta Region AIDS Education & Training Center* 2004; **16**(2): 1-3.

44. Patten GE, Wilkinson L, Conradie K, et al. Impact on ART initiation of point-of-care CD4 testing at HIV diagnosis among HIV-positive youth in Khayelitsha, South Africa. *Journal of the International AIDS Society* 2013; **16**: 18518.

45. Puccio JA, Belzer M, Olson J, et al. The use of cell phone reminder calls for assisting HIV-infected adolescents and young adults to adhere to highly active antiretroviral therapy: A pilot study. *AIDS patient care and STDs* 2006; **20**(6): 438-44.

46. Purdy JB, Freeman AF, Martin SC, et al. Virologic response using directly observed therapy in adolescents with HIV: an adherence tool. *The Journal of the Association of Nurses in AIDS Care : JANAC* 2008; **19**(2): 158-65.

47. Rawlings MK, Thompson MA, Farthing CF, et al. Impact of an educational program on efficacy and adherence with a twice-daily lamivudine/zidovudine/abacavir regimen in underrepresented HIV-infected patients. *Journal of acquired immune deficiency syndromes (1999)* 2003; **34**(2): 174-83.

48. Reisner SL, Mimiaga MJ, Skeer M, Perkovich B, Johnson CV, Safren SA. A review of HIV antiretroviral adherence and intervention studies among HIV-infected youth. *Topics in HIV medicine : a publication of the International AIDS Society, USA* 2009; **17**(1): 14-25.

49. Reynolds E, Berrien V, Acosta-Glynn C, Salazar JC. Home based, intense, nursing intervention trial improves adherence to HAART in HIV infected children. *Pediatric Research* 2001; **49**(4 Part 2): 252A-A.

50. Rogers AS, Miller S, Murphy DA, Tanney M, Fortune T. The TREAT (Therapeutic Regimens Enhancing Adherence in Teens) program: theory and preliminary results. *The Journal of adolescent health : official publication of the Society for Adolescent Medicine* 2001; **29**(3 Suppl): 30-8.

51. Rongkavilit C, Naar-King S, Wang B, et al. Motivational interviewing targeting risk behaviors for youth living with HIV in Thailand. *AIDS Behav* 2013; **17**(6): 2063-74.

52. Rongkavilit C, Naar-King S, Koken JA, et al. A feasibility study of motivational interviewing for health risk behaviors among Thai youth living with HIV. *The Journal of the Association of Nurses in AIDS Care : JANAC* 2014; **25**(1): 92-7.

53. Rotheram-Borus MJ, Swendeman D, Comulada WS, Weiss RE, Lee M, Lightfoot M. Prevention for substance-using HIV-positive young people: telephone and in-person delivery. *Journal of acquired immune deficiency syndromes (1999)* 2004; **37 Suppl 2**: S68-77.

54. Rueda S, Park-Wyllie LY, Bayoumi AM, et al. Patient support and education for promoting adherence to highly active antiretroviral therapy for HIV/AIDS. *Cochrane database of systematic reviews (Online)* 2006; **3**.

55. Saberi P, Yuan P, John M, Sheon N, Johnson MO. A pilot study to engage and counsel HIV-positive African American youth via telehealth technology. *AIDS patient care and STDs* 2013; **27**(9): 529-32.

56. Saberi P, Mayer K, Vittinghoff E, Naar-King S. Correlation Between Use of Antiretroviral Adherence Devices by HIV-Infected Youth and Plasma HIV RNA and Self-Reported Adherence. *AIDS Behav* 2014.

57. Shegog R, Markham CM, Leonard AD, Bui TC, Paul ME. "+CLICK": pilot of a web-based training program to enhance ART adherence among HIV-positive youth. *AIDS care* 2012; **24**(3): 310-8.

58. Simoni JM, Huh D, Frick PA, et al. Peer support and pager messaging to promote antiretroviral modifying therapy in seattle: A randomized controlled trial. *Journal of Acquired Immune Deficiency Syndromes* 2009; **52**(4): 465-73.

59. Snyder K, Wallace M, Duby Z, et al. Preliminary results from Hlanganani (Coming Together): A structured support group for HIV-infected adolescents piloted in Cape Town, South Africa. *Children and Youth Services Review* 2014.

60. Van Der Plas A, Scherpbier H, Kuijpers T, Pajkrt D. The effect of different intervention programs on treatment adherence of HIV-infected children, a retrospective study. *AIDS Care - Psychological and Socio-Medical Aspects of AIDS/HIV* 2013; **25**(6): 738-43.

61. Van Winghem J, Telfer B, Reid T, et al. Implementation of a comprehensive program including psycho-social and treatment literacy activities to improve adherence to HIV care and treatment for a pediatric population in Kenya. *BMC Pediatrics* 2008; **8**.

62. Wohl AR, Garland WH, Wu J, et al. A youth-focused case management intervention to engage and retain young gay men of color in HIV care. *AIDS Care - Psychological and Socio-Medical Aspects of AIDS/HIV* 2011; **23**(8): 988-97.

63. Wynberg E, Cooke G, Shroufi A, Reid SD, Ford N. Impact of point-of-care CD4 testing on linkage to HIV care: a systematic review. *Journal of the International AIDS Society* 2014; **17**: 18809.

64. Berrien VM, Salazar JC, Reynolds E, McKay K. Adherence to antiretroviral therapy in HIV-infected pediatric patients improves with home-based intensive nursing intervention. *AIDS patient care and STDs* 2004; **18**(6): 355-63.

65. Bhana A, Mellins CA, Petersen I, et al. The VUKA family program: piloting a family-based psychosocial intervention to promote health and mental health among HIV infected early adolescents in South Africa. *AIDS care* 2014; **26**(1): 1-11.

66. Letourneau EJ, Ellis DA, Naar-King S, Chapman JE, Cunningham PB, Fowler S. Multisystemic therapy for poorly adherent youth with HIV: results from a pilot randomized controlled trial. *AIDS care* 2013; **25**(4): 507-14.

67. Davila JA, Miertschin N, Sansgiry S, Schwarzwald H, Henley C, Giordano TP. Centralization of HIV services in HIV-positive African-American and Hispanic youth improves retention in care. *AIDS care* 2013; **25**(2): 202-6.

68. Foster C, McDonald S, Frize G, Ayers S, Fidler S. "Payment by Results"--financial incentives and motivational interviewing, adherence interventions in young adults with perinatally acquired HIV-1 infection: a pilot program. *AIDS patient care and STDs* 2014; **28**(1): 28-32.

69. Funck-Brentano I, Dalban C, Veber F, et al. Evaluation of a peer support group therapy for HIV-infected adolescents. *AIDS (London, England)* 2005; **19**(14): 1501-8.

70. Glikman D, Walsh L, Valkenburg J, Mangat PD, Marcinak JF. Hospital-based directly observed therapy for HIV-infected children and adolescents to assess adherence to antiretroviral medications. *Pediatrics* 2007; **119**(5): e1142-8.

71. Kaihin R, Kasatpibal N, Chitreechuer J, Grimes RM. Effect of an Empowerment Intervention on Antiretroviral Drug Adherence in Thai Youth. *Behavioral medicine (Washington, DC)* 2014: 0.

72. Lamb MR, Fayorsey R, Nuwagaba-Biribonwoha H, et al. High attrition before and after ART initiation among youth (15-24 years of age) enrolled in HIV care. *AIDS (London, England)* 2014; **28**(4): 559-68.

73. Lyon ME, Trexler C, Akpan-Townsend C, et al. A family group approach to increasing adherence to therapy in HIV-infected youths: results of a pilot project. *AIDS patient care and STDs* 2003; **17**(6): 299-308.

74. Parsons GN, Siberry GK, Parsons JK, et al. Multidisciplinary, inpatient directly observed therapy for HIV-1-infected children and adolescents failing HAART: A retrospective study. *AIDS patient care and STDs* 2006; **20**(4): 275-84.
